# Histamine-2 receptor antagonists versus proton pump inhibitors for septic shock after lower gastrointestinal tract perforation: a retrospective cohort study using a national inpatient database

**DOI:** 10.1186/s40560-020-00473-0

**Published:** 2020-07-31

**Authors:** Jun Suzuki, Yusuke Sasabuchi, Shuji Hatakeyama, Hiroki Matsui, Teppei Sasahara, Yuji Morisawa, Toshiyuki Yamada, Hideo Yasunaga

**Affiliations:** 1 Division of Infectious Diseases, Jichi Medical University Hospital, 3311-1 Yakushiji, Shimotsuke, Tochigi, 329-0498 Japan; 2 Data Science Center, Jichi Medical University, 3311-1 Yakushiji, Shimotsuke, Tochigi, 329-0498 Japan; 3 Division of General Medicine, Jichi Medical University, 3311-1 Yakushiji, Shimotsuke, Tochigi, 329-0498 Japan; 4 Department of Clinical Epidemiology and Health Economics, School of Public Health, The University of Tokyo, 7-3-1 Hongo, Bunkyo-ku, Tokyo, 113-0033 Japan; 5 Department of Infection and Immunity, School of Medicine, Jichi Medical University, 3311-1 Yakushiji, Shimotsuke, Tochigi, 329-0498 Japan; 6 Department of Clinical Laboratory Medicine, Jichi Medical University, 3311-1 Yakushiji, Shimotsuke, Tochigi, 329-0498 Japan

**Keywords:** Bleeding, *Clostridioides difficile* infection, Histamine-2 receptor antagonists, Mortality, Hospital-acquired pneumonia, Proton pump inhibitors, Peritonitis

## Abstract

**Background:**

Studies have shown the potential benefit of stress ulcer prophylaxis including histamine-2 receptor antagonists (H2RA) and proton pump inhibitors (PPI) in critically ill patients. However, the adverse effects of stress ulcer prophylaxis such as *Clostridioides difficile* infection (CDI) and hospital-acquired pneumonia have been reported. Abdominal septic shock is associated with increased risk of bleeding, CDI, and pneumonia; however, which ulcer prophylaxis might be associated with better outcomes in patients with septic shock after lower gastrointestinal tract perforation is unknown.

**Methods:**

In this retrospective cohort study using the Japanese Diagnosis Procedure Combination database from July 2010 to March 2015, we identified patients aged 18 years or older who received open abdominal surgery for lower gastrointestinal tract perforation and who used vasopressors and antibiotics within 2 days of admission. We performed propensity score matching and inverse probability of treatment weighting (IPTW) to compare the outcomes between patients who received H2RA and those who received PPI within 2 days of admission. The outcomes included gastrointestinal bleeding requiring endoscopic hemostasis within 28 days of admission, 28-day mortality, CDI, and hospital-acquired pneumonia.

**Results:**

The propensity score matching created 1088 pairs of patients who received H2RA or PPI within 2 days of admission. There were no significant differences between the H2RA and PPI groups regarding gastrointestinal bleeding requiring endoscopic hemostasis within 28 days of admission (0.74% vs 1.3%, risk ratio 0.57 (0.24–1.4), and *P* = 0.284), 28-day mortality (11.3% vs 12.9%, risk ratio 0.88 (0.68–1.1), and *P* = 0.386), CDI (0.64% vs 0.46%, risk ratio 1.4 (0.45–4.4), and *P* = 0.774), and hospital-acquired pneumonia (3.0% vs 4.3%, risk ratio 0.70 (0.45–1.1), and *P* = 0.138). IPTW analysis showed similar results.

**Conclusions:**

There were no significant differences in gastrointestinal bleeding requiring endoscopic hemostasis within 28 days of admission, 28-day mortality, CDI, and hospital-acquired pneumonia between H2RA and PPI in patients with septic shock after lower gastrointestinal tract perforation.

## Background

Septic shock after lower gastrointestinal tract perforation is one of major causes of abdominal infection, and the mortality has been reported to be 18 to 50% [1–3]. *Clostridioides difficile* (formerly *Clostridium difficile*) infection (CDI) and pneumonia are common complications after abdominal infection [4–6], and these complications are associated with higher mortality in patients with septic shock after lower gastrointestinal tract perforation [7, 8].

Histamine-2 receptor antagonists (H2RA) and proton pump inhibiter (PPI) play an essential role in stress ulcer prophylaxis in patients with septic shock [9, 10]. There are several concerns that stress ulcer prophylaxis was associated with CDI [11] and hospital-acquired pneumonia [12]. Previous systematic review and meta-analysis suggested that PPI and H2RA showed similar risk of hospital-acquired pneumonia or CDI [13, 14]. However, clinical heterogeneity between studies included in these meta-analyses was substantial. On the other hand, a retrospective cohort study suggested that the frequency of adverse effects of H2RA may differ from those of PPI [14]. If the risk of CDI or hospital-acquired pneumonia is different, a lower-risk drug should be selected. However, it is not known whether PPI and H2RA show similar risk of hospital-acquired pneumonia and CDI for patients with septic shock due to lower gastrointestinal tract perforation.

Therefore, the purpose of this study was to compare H2RA and PPI with regard to gastrointestinal bleeding, 28-day mortality, CDI, and hospital-acquired pneumonia for patients with septic shock after lower gastrointestinal tract perforation using a Japanese national inpatient database.

## Methods

### Data source

Data for this study were extracted from the Japanese Diagnosis Procedure Combination database, which is a nationwide administrative claims database with discharge abstracts representing approximately 50% of all admissions to acute care hospitals in Japan. The database includes the following data: (1) patient demographic data; (2) primary diagnosis, comorbidities at admission, post-admission complications during hospitalization coded with the *International Classification of Diseases, 10th revision* (ICD-10) and text written in Japanese language; (3) hospital identification number; (4) dates of surgery, procedures, and drug prescription; (5) discharge status (dead or alive); and (6) dates of hospital admission and discharge [15–17].

### Patient data

We identified patients with septic shock after lower gastrointestinal tract perforation hospitalized between July 2010 and March 2015. We included patients who (1) were diagnosed with septic shock, (2) diagnosed with lower gastrointestinal tract perforation, (3) required open abdominal surgery within 2 days of admission, and (4) used antibiotics within 2 days of admission. Sepsis was defined as having any bacterial or fungal infection at admission based on the Angus criteria [1] (Additional Table 1). Definition of sepsis based on the Angus criteria has been validated in several DPC hospitals in Japan (the sensitivity value of 40.4, the specificity value of 83.0, and the positive predictive value of 79.8% for sepsis) [18]. Septic shock was defined as combination of diagnosis of sepsis and requirement of vasopressors within 2 days of admission [19]. Lower gastrointestinal tract perforation was identified with ICD-10 codes (K65.0, K63.1, K57.02, K57.03, K57.22, K57.23, K57.42, K57.43, K57.82, and K57.83) in the primary diagnosis or comorbidities at admission. We included patients who underwent open abdominal surgery within 2 days of admission because patients sometimes receive surgery on the next day if a patient is admitted late at night. Exclusion criteria were as follows [12]: (1) age < 18 years, (2) discharge within 2 days of admission, (3) pregnancy, (4) human immunodeficiency virus infection or acquired immunodeficiency syndrome, (5) sucralfate use within 2 days of admission, (6) medical history of peptic ulcer, (7) anticoagulant or antiplatelet drug use within 2 days of admission, (8) neither H2RA nor PPI used within 2 days of admission, and (9) both H2RA and PPI used within 2 days of admission.

### Study variables

The H2RA group was defined as patients who received H2RA within 2 days of admission, whereas the PPI group was defined as patients who received PPI within 2 days of admission. Other variables included age, sex, ICU admission within 2 days of admission, high care unit (HCU) admission within 2 days of admission, hospital type (academic or not), hospital volume, and Japan coma scale (JCS). Age was categorized at 10-year intervals. Hospital volume was defined as the annual mean number of patients with lower gastrointestinal tract perforation requiring open abdominal surgery. The JCS score was recorded in all patients to assess the level of consciousness on admission, and it correlated well with the Glasgow Coma Scale [20]. JCS scores were divided into 4 categories: 0 (alert), 1–3 (delirium), 10–30 (somnolence), and 100–300 (coma) [20, 21]. The use of the following procedures within 2 days of admission were also evaluated: mechanical ventilation, intermittent and continuous renal replacement therapy, polymyxin B hemoperfusion, central venous catheter insertion, vasopressor use, transfusions (red cells, platelet concentrates, fresh-frozen plasma), antithrombin, recombinant human soluble thrombomodulin, immunoglobulin, danaparoid, hydrocortisone, primary use of antibiotics (penicillin, ampicillin, ampicillin/sulbactam, piperacillin/tazobactam, first-generation cephalosporin, second-generation cephalosporin, third-generation cephalosporin with or without effects for *Pseudomonas aeruginosa*, fourth-generation cephalosporin, carbapenem, aminoglycoside, fluoroquinolone, clindamycin, macrolide, tetracycline, anti-methicillin-resistant *Staphylococcus aureus* drugs and antifungal drugs), and two or more classes of initial antibiotic combinations.

### Outcomes

Outcomes of interest in this study were gastrointestinal bleeding (ICD-10 code: K25, K26, K27, K28, K29, K920, and K922) requiring endoscopic hemostasis within 28 days of admission, 28-day mortality, CDI (ICD-10 code: A047) coded as a complication during hospitalization, and hospital-acquired pneumonia.

### Statistical analysis

Descriptive statistics were presented before and after propensity score matching. Continuous variables were presented as the mean with standard deviation (SD). Categorical variables are presented as numbers with percentages.

One-to-one propensity score matching was used to adjust for differences in baseline characteristics and the severity of condition at admission between the H2RA and PPI groups. The probability that a patient received H2RA was modeled for confounders for the following characteristics: age, age category, sex, hospital type, hospital volume, comorbidities at admission, use of mechanical ventilation, intermittent and continuous renal replacement therapy, polymyxin B hemoperfusion, central venous catheter insertion, vasopressor use, transfusion, antithrombin, recombinant human soluble thrombomodulin, immunoglobulin, low molecular heparin, danaparoid, hydrocortisone, primary use of antibiotics, and use of two or more initial antibiotics. Differences between the H2RA and PPI groups before and after propensity score matching were assessed by standardized mean differences [22]. Absolute standardized mean differences of less than 0.1 were considered as negligible imbalances in the baseline characteristics between the groups [23]. Fisher’s exact test was used to compare outcomes between the two groups. We also estimated the treatment effect using inverse probability of treatment weighting (IPTW) using propensity scores. We calculated risk ratio, risk differences, and their 95% confidence intervals (CI) between unmatched, propensity-matched, and IPTW analyses [24]. A *P* value of less than 0.05 was considered statistically significant. Propensity score matching was performed using the “matching” package in the statistical software R version 3.1.3 (The R Foundation, Vienna, Austria). IPTW analyses were performed using the “survey” package in the statistical software R version 3.1.3. All other analyses were performed using the IBM SPSS software version 25 (IBM SPSS, Armonk, NY).

## Results

**Fig. 1:**
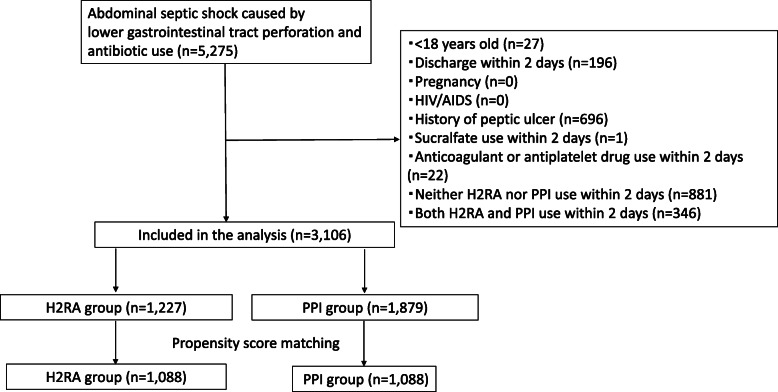
Flow chart for patient inclusion. *Abbreviations*: H2RA, histamine-2 receptor antagonists; PPI, proton pump inhibiter; HIV, human, human immunodeficiency virus; AIDS, acquired immunodeficiency syndrome

Overall, 3106 patients were identified during the study period. The H2RA group included 1227 patients, and the PPI group included 1879 patients. After one-to-one propensity score matching, 1088 pairs were created (Fig. 1).

Table 1 shows the baseline characteristics of the unmatched (*n* = 3106) and the propensity score-matched groups (*n* = 2176). After propensity score matching, the patients’ backgrounds were well-balanced between the H2RA and PPI groups.

**Table 1:** Baseline patient characteristics in unmatched and propensity-matched groups

Variables	Unmatched group			Matched group		
	H2RA group	PPI group	SMD	H2RA group	PPI group	SMD
	*n* = 1227	*n* = 1879		*n* = 1088	*n* = 1088	
Age, mean (SD)	74.5 (13.1)	73.7 (13.0)	0.06	74.2 (13.2)	74.4 (12.9)	0.02
Age category			0.09			0.06
10–19	1 (0.1)	0 (0.0)		1 (0.1)	0 (0.0)	
20–29	7 (0.7)	6 (0.5)		5 (0.5)	4 (0.4)	
30–39	19 (1.8)	23 (2.0)		19 (1.7)	19 (1.7)	
40–49	30 (2.8)	39 (3.4)		31 (2.8)	27 (2.5)	
50–59	91 (8.6)	96 (8.5)		82 (7.5)	88 (8.1)	
60–69	198 (18.7)	207 (18.3)		196 (18.0)	196 (18.0)	
70–79	324 (30.6)	342 (30.2)		308 (28.3)	301 (27.7)	
80–89	326 (30.8)	365 (32.3)		364 (33.5)	366 (33.6)	
90–99	62 (5.9)	52 (4.6)		80 (7.4)	85 (7.8)	
100–	0 (0.0)	1 (0.1)		2 (0.2)	2 (0.2)	
Sex (female), *n* (%)	615 (50.1)	891 (47.4)	0.05	545 (50.1)	542 (49.8)	0.01
ICU admission, *n* (%)	449 (36.6)	867 (46.1)	0.20	414 (38.1)	434 (39.9)	0.04
HCU admission, *n* (%)	52 (4.2)	99 (5.3)	0.05	50 (4.6)	50 (4.6)	< 0.001
Hospital type (academic center), *n* (%)	240 (19.6)	606 (32.3)	0.29	239 (22.0)	206 (18.9)	0.08
Hospital volume, case/year mean (SD)	5.5 (4.9)	7.2 (7.4)	0.27	5.7 (5.1)	5.7 (5.2)	0.01
**Comorbidity,*n*(%)**						
Myocardial infarction	16 (1.3)	17 (0.9)	0.04	11 (1.0)	9 (0.8)	0.02
Congestive heart failure	70 (5.7)	130 (6.9)	0.05	65 (6.0)	58 (5.3)	0.03
Peripheral vascular disease	16 (1.3)	26 (1.4)	0.01	14 (1.3)	13 (1.2)	0.01
Cerebrovascular disease	58 (4.7)	72 (3.8)	0.04	47 (4.3)	46 (4.2)	0.01
Dementia	34 (2.8)	48 (2.6)	0.01	28 (2.6)	27 (2.5)	0.01
Chronic pulmonary disease	41 (3.3)	52 (2.8)	0.03	30 (2.8)	36 (3.3)	0.03
Mild liver disease	37 (3.0)	70 (3.7)	0.04	33 (3.0)	32 (2.9)	0.01
Severe liver disease	1 (0.1)	7 (0.4)	0.06	1 (0.1)	0 (0.0)	0.04
Rheumatologic disease	30 (2.4)	42 (2.2)	0.01	25 (2.3)	24 (2.2)	0.006
Hemiplegia or paraplegia	2 (0.2)	3 (0.2)	0.001	2 (0.2)	1 (0.1)	0.03
Diabetes without chronic complications	120 (9.8)	182 (9.7)	0.003	104 (9.6)	95 (8.7)	0.03
Diabetes with chronic complications	23 (1.9)	43 (2.3)	0.03	20 (1.8)	21 (1.9)	0.007
Renal disease	71 (5.8)	183 (9.7)	0.15	69 (6.3)	69 (6.3)	< 0.001
Any malignancy, including leukemia and lymphoma	207 (16.9)	300 (16.0)	0.02	188 (17.3)	184 (16.9)	0.01
Metastatic solid tumor	66 (5.4)	96 (5.1)	0.01	61 (5.6)	58 (5.3)	0.01
**Consciousness level**, *n* (%)						
Alert	925 (75.4)	1308 (69.6)	0.13	808 (74.3)	813 (74.7)	0.01
Delirium	178 (14.5)	324 (17.2)	0.08	167 (15.3)	166 (15.3)	0.003
Somnolence	50 (4.1)	100 (5.3)	0.06	43 (4.0)	48 (4.4)	0.02
Coma	55 (4.5)	115 (6.1)	0.07	53 (4.9)	48 (4.4)	0.02
**Intervention**, *n* (%)						
Mechanical ventilation	667 (54.4)	1258 (67.0)	0.26	625 (57.4)	638 (58.6)	0.02
Intermittent renal replacement therapy	176 (14.3)	364 (19.4)	0.14	170 (15.6)	177 (16.3)	0.02
Continuous renal replacement therapy	23 (1.9)	64 (3.4)	0.10	21 (1.9)	21 (1.9)	< 0.001
Polymyxin B hemoperfusion	306 (24.9)	552 (29.4)	0.10	284 (26.1)	284 (26.1)	< 0.001
Central venous insertion	350 (28.5)	783 (41.7)	0.28	335 (30.8)	345 (31.7)	0.02
**Catecholamine**, *n* (%)						
Dopamine	948 (77.3)	1213 (64.6)	0.28	809 (74.4)	820 (75.4)	0.02
Noradrenaline	660 (53.8)	1324 (70.5)	0.35	641 (58.9)	631 (58.0)	0.02
Vasopressin	47 (3.8)	143 (7.6)	0.16	47 (4.3)	47 (4.3)	< 0.001
Adrenaline	69 (5.6)	122 (6.5)	0.04	65 (6.0)	68 (6.2)	0.01
**Transfusion,*n*(%)**						
Red cell transfusion	379 (30.9)	733 (39.0)	0.17	361 (33.2)	369 (33.9)	0.02
Platelets transfusion	54 (4.4)	142 (7.6)	0.13	52 (4.8)	58 (5.3)	0.03
Fresh frozen plasma transfusion	327 (26.7)	471 (25.1)	0.04	288 (26.5)	292 (26.8)	0.01
Antithrombin, *n* (%)	245 (20.0)	463 (24.6)	0.11	234 (21.5)	232 (21.3)	0.004
Recombinant human soluble thrombomodulin, *n* (%)	198 (16.1)	383 (20.4)	0.11	190 (17.5)	192 (17.6)	0.005
Immunoglobulin, *n* (%)	457 (37.2)	735 (39.1)	0.04	428 (39.3)	393 (36.1)	0.07
Albumin, *n* (%)	823 (67.1)	1369 (72.9)	0.13	738 (67.8)	753 (69.2)	0.03
Danaparoid, *n* (%)	6 (0.5)	22 (1.2)	0.08	6 (0.6)	10 (0.9)	0.04
Low-molecular-weight heparin, *n* (%)	11 (0.9)	27 (1.4)	0.05	11 (1.0)	14 (1.3)	0.03
Hydrocortisone, *n* (%)	155 (12.6)	340 (18.1)	0.15	145 (13.3)	145 (13.3)	< 0.001
**Initial antibiotics use**, *n* (%)						
Initial use of two or more	342 (27.9)	568 (30.2)	0.05	320 (29.4)	329 (30.2)	0.02
Penicillin	0 (0.0)	2 (0.1)	0.05	0 (0.0)	0 (0.0)	< 0.001
Ampicillin	0 (0.0)	2 (0.1)	0.05	0 (0.0)	0 (0.0)	< 0.001
Ampicillin/sulbactam	27 (2.2)	46 (2.4)	0.02	25 (2.3)	27 (2.5)	0.01
Piperacillin/tazobactam	88 (7.2)	217 (11.5)	0.15	86 (7.9)	97 (8.9)	0.04
First-generation cephalosporin	47 (3.8)	66 (3.5)	0.02	42 (3.9)	45 (4.1)	0.01
Second-generation cephalosporin	499 (40.7)	573 (30.5)	0.21	418 (38.4)	419 (38.5)	0.002
Third-generation cephalosporin without effect for *Pseudomonas aeruginosa*	43 (3.5)	53 (2.8)	0.04	40 (3.7)	41 (3.8)	0.01
Third-generation cephalosporin with effect for *Pseudomonas aeruginosa*	3 (0.2)	8 (0.4)	0.03	3 (0.3)	3 (0.3)	<0.001
Fourth-generation cephalosporin	30 (2.4)	44 (2.3)	0.01	29 (2.7)	27 (2.5)	0.01
Carbapenem	753 (61.4)	1306 (69.5)	0.17	703 (64.6)	690 (63.4)	0.03
Fluoroquinolone	7 (0.6)	11 (0.6)	0.002	6 (0.6)	5 (0.5)	0.01
Aminoglycoside	18 (1.5)	18 (1.0)	0.05	16 (1.5)	13 (1.2)	0.02
Clindamycin	50 (4.7)	40 (3.5)	0.06	41 (3.8)	45 (4.1)	0.02
Tetracycline	1 (0.1)	5 (0.3)	0.04	1 (0.1)	2 (0.2)	0.03
Macrolide	0 (0.0)	7 (0.4)	0.09	0 (0.0)	0 (0.0)	< 0.001
Anti-MRSA drug	20 (1.6)	62 (3.3)	0.11	20 (1.8)	20 (1.8)	< 0.001
Antifungal drug	4 (0.3)	14 (0.7)	0.06	4 (0.4)	5 (0.5)	0.02

*H2RA* histamine-2 receptor antagonists, *PPI* proton pump inhibiter, *SD* standard deviation, *ICU* intensive care unit, *HCU* high care unit, *MRSssA* methicillin-resistant *Staphylococcus aureus*, *SMD* standardized mean difference

Table 2 shows the outcomes of the two groups. Before propensity score matching, there were no significant differences for gastrointestinal bleeding requiring endoscopic hemostasis, 28-day mortality, CDI, and hospital-acquired pneumonia between the groups (Table 2).

**Table 2:** Outcomes in the unmatched and propensity-matched groups

	Unmatched groups			Propensity-matched groups		
	H2RA group	PPI group	*P*	H2RA group	PPI group	*P*
Outcome, *n* (%)	*n* = 1227	*n* = 1879		*n* = 1088	*n* = 1088	
Gastrointestinal bleeding requiring endoscopic hemostasis	8 (0.65)	25 (1.33)	0.076	8 (0.74)	14 (1.3)	0.284
28-day mortality	130 (10.6)	234 (12.4)	0.954	123 (11.3)	141 (12.9)	0.386
*Clostridioides difficile* infection	10 (0.81)	12 (0.64)	0.663	7 (0.64)	5 (0.46)	0.774
Hospital-acquired pneumonia	38 (3.1)	64 (3.4)	0.681	33 (3.0)	47 (4.3)	0.138

*H2RA* histamine-2 receptor antagonists, *PPI* proton pump inhibiter

After propensity score matching, there were no significant differences between the groups for gastrointestinal bleeding requiring endoscopic hemostasis within 28 days of admission (0.74% vs 1.3%, *P* = 0.284), 28-day mortality (11.3% vs 12.9%, *P* = 0.386), CDI (0.64% vs 0.46%, *P* = 0.774), and hospital-acquired pneumonia (3.0% vs 4.3%, *P* = 0.138) (Table 2). Risk differences in the unmatched, propensity score-matched, and IPTW analysis groups showed similar results (Additional Table 2 and additional Table 3).

Table 3 shows risk ratios of the two groups. Before and after propensity score matching, there were no significant differences for gastrointestinal bleeding requiring endoscopic hemostasis, 28-day mortality, CDI, and hospital-acquired pneumonia between the groups (Table 3).

**Table 3:** Risk ratios in the unmatched and propensity-matched groups

Outcomes	Unmatched groups (95% CI)	Propensity-matched groups (95% CI)
Gastrointestinal bleeding requiring endoscopic hemostasis	0.49 (0.22–1.08)	0.57 (0.24–1.36)
28-day mortality	0.85 (0.70–1.04)	0.87 (0.70–1.09)
*Clostridioides difficile* infection	1.28 (0.55–2.94)	1.40 (0.45–4.40)
Hospital-acquired pneumonia	0.91 (0.61–1.35)	0.70 (0.45–1.09)

*CI* confidence interval

## Discussion

In this retrospective study using a national inpatient database of Japan, there were no significant differences in gastrointestinal bleeding requiring endoscopic hemostasis within 28 days of admission, 28-day mortality, CDI, and hospital-acquired pneumonia between the H2RA and PPI groups in patients with septic shock after lower gastrointestinal perforation.

It is controversial whether H2RA increases gastrointestinal bleeding compared with PPI [13, 14]. The effect of pharmacological acid suppression of H2RA was reported to be lower than that of PPI for treating active gastrointestinal bleeding [25]. However, H2RA reaches the target pH for stress ulcer prophylaxis within a day in approximately 65% of patients [26] and may be sufficient for stress ulcer prophylaxis. Thus, gastrointestinal bleeding requiring endoscopic hemostasis was not different between the groups.

It is unknown whether H2RA increases the risk of CDI compared with PPI. Gastric acid functions as a physiological barrier; however, H2RA and PPI altered these barrier mechanisms and were associated with bacterial overgrowth [27]. However, Clostridium species are usually acid-resistant and cells remain viable at gastric pH levels [28]; therefore, H2RA and/or PPI may not be associated with the increased risk of CDI. These points may also explain our results.

It is controversial whether H2RA increases the risk of hospital-acquired pneumonia compared with PPI. A gastric pH > 4 was associated with bacterial overgrowth and colonization and was associated with hospital-acquired pneumonia [29]. Although the pharmacological acid suppression of H2RA may be lower than that of PPI, both H2RA and PPI induced pH > 4 within 1 day of administration [26]. Therefore, the frequency of hospital-acquired pneumonia by H2RA may be similar to that of PPI.

Our study had several strengths. To the best of our knowledge, it is the first study to evaluate the effect of H2RA compared with PPI in patients with septic shock after lower gastrointestinal tract perforation. Second, our study design was based on a real-world clinical setting and included approximately 50% of inpatients who were admitted to acute-care hospitals in Japan.

Our study had several limitations. First, the database lacks laboratory data such as lactate and clinical records including the results of cultures and susceptibility to peritonitis pathogens. Second, in Japan, patients with septic shock after lower gastrointestinal tract perforation were treated on general wards, and our results may not be generalized to other countries. Third, the present study could not assess mild-to-moderate gastrointestinal bleeding without requiring endoscopic hemostasis because the database did not include information regarding this status. Fourth, the database does not include information about initiation time of drug administration. Several procedures or drug administrations might have been performed before H2RA and PPI administration. Fifth, 28-day mortality in the present study was relatively low compared to those in previous studies. The difference may be due to exclusion of patients who died within 2 days of admission in the present study. Sixth, CDI or hospital-acquired pneumonia might have been underestimated, which could have led to less statistical power. Seventh, we defined sepsis using Angus criteria, which was validated in the DPC database. Although specificity of this definition was high, low sensitivity of sepsis may preclude extrapolation to other population. Last, although we used propensity score matching to adjust for patient backgrounds, unmeasured confounding factors might have biased our results.

## Conclusions

In our study, H2RA were not associated with CDI, gastrointestinal bleeding requiring endoscopic hemostasis, 28-day mortality, or hospital-acquired pneumonia compared with PPI in patients with septic shock after lower gastrointestinal tract perforation.

## Supplementary information

**Additional file 1: Additional Table 1.** ICD-10 codes to define sepsis.

**Additional file 2: Additional Table 2.** Patient characteristsics in the IPTW analysis group.

**Additional file 3: Additional Table 3.** Risk differences for outcomes in the unmatched, propensity score-matched, and IPTW analysis groups.

## Data Availability

Data cannot be made publicly available for ethical reasons because the data contains patient information. The study data are available to interested researchers upon reasonable request to the corresponding author, pending ethical approval.

## Abbreviations

AIDS: Acquired immunodeficiency syndrome
CDI: *Clostridioides difficile* infection
CI: Confidence interval
HCU: High care unit
HIV: Human immunodeficiency virus
H2RA: Histamine-2 receptor antagonists
ICD-10: International Classification of Diseases, Tenth Revision
IPTW: Inverse probability of treatment weighting
MRSA: Methicillin-resistant *Staphylococcus aureus*
PPI: Proton pump inhibitor
SD: Standard deviation
SMD: Standardized mean difference
