# Supplementary material for: Histamine-2 receptor antagonists versus proton pump inhibitors for septic shock after lower gastrointestinal tract perforation: a retrospective cohort study using a national inpatient database
Source: J Intensive Care. 2020 Jul 31;8:56. doi: 10.1186/s40560-020-00473-0 (PMC7395359; doi:10.1186/s40560-020-00473-0)
Supplement: Supplementary file 2 — Additional file 2: Additional Table 2. Patient characteristsics in the IPTW analysis group. [file 40560_2020_473_MOESM2_ESM.docx]

**Additional Table 2. Patient characteristics in the IPTW analysis group.**

|  | **IPTW analysis group** | | |
| --- | --- | --- | --- |
|  | H2RA group | PPI group | SMD |
| **Variables** | n=3,085 | n=3,113 |  |
| Age, mean (SD) | 74.0 (13.2) | 74.1 (13.0) | 0.01 |
| Age category |  |  | 0.04 |
| 10-19 | 2 (0.0) | 2 (0.0) |  |
| 20-29 | 15 (0.5) | 16 (0.5) |  |
| 30-39 | 50 (1.6) | 51 (1.6) |  |
| 40-49 | 79 (2.6) | 87 (2.8) |  |
| 50-59 | 278 (9.0) | 251 (8.1) |  |
| 60-69 | 543 (17.6) | 553 (17.8) |  |
| 70-79 | 866 (28.1) | 890 (28.6) |  |
| 80-89 | 1037 (33.6) | 1053 (33.8) |  |
| 90-99 | 211 (6.8) | 208 (6.7) |  |
| 100- | 4 (0.1) | 4 (0.1) |  |
| Sex (female), n (%) | 1513 (49.0) | 1525 (49.0) | 0.001 |
| ICU admission, n (%) | 1321 (42.8) | 1318 (42.3) | 0.01 |
| HCU admission, n (%) | 161 (5.2) | 155 (5.0) | 0.01 |
| Hospital type (academic center), n (%) | 828 (26.8) | 839 (27.0) | 0.003 |
| Hospital volume, case/year mean (SD) | 6 (5.8) | 6 (6.3) | 0.01 |
| **Comorbidity, n (%)** |  |  |  |
| Myocardial infarction | 27 (0.9) | 28 (0.9) | 0.01 |
| Congestive heart failure | 193 (6.2) | 196 (6.3) | 0.002 |
| Peripheral vascular disease | 40 (1.3) | 41 (1.3) | 0.003 |
| Cerebrovascular disease | 119 (3.9) | 128 (4.1) | 0.01 |
| Dementia | 78 (2.5) | 81 (2.6) | 0.01 |
| Chronic pulmonary disease | 86 (2.8) | 88 (2.8) | 0.002 |
| Mild liver disease | 95 (3.1) | 106 (3.4) | 0.02 |
| Severe liver disease | 11 (0.4) | 8 (0.3) | 0.02 |
| Rheumatologic disease | 84 (2.7) | 72 (2.3) | 0.03 |
| Hemiplegia or paraplegia | 4 (0.1) | 5 (0.1) | 0.004 |
| Diabetes without chronic complications | 291 (9.4) | 297 (9.5) | 0.004 |
| Diabetes with chronic complications | 68 (2.2) | 65 (2.1) | 0.01 |
| Renal disease | 246 (8.0) | 254 (8.2) | 0.01 |
| Any malignancy, including leukemia and lymphoma | 518 (16.8) | 510 (16.4) | 0.01 |
| Metastatic solid tumor | 169 (5.5) | 165 (5.3) | 0.01 |
| **Consciousness level**, *n* (%) |  |  |  |
| Alert | 2258 (73.2) | 2248 (72.2) | 0.02 |
| Delirium | 471 (15.3) | 496 (15.9) | 0.02 |
| Somnolence | 135 (4.4) | 146 (4.7) | 0.02 |
| Coma | 172 (5.6) | 171 (5.5) | 0.004 |
| **Intervention**, *n* (%) |  |  |  |
| Mechanical ventilation | 1900 (61.6) | 1923 (61.8) | 0.004 |
| Intermittent renal replacement therapy | 552 (17.9) | 549 (17.6) | 0.01 |
| Continuous renal replacement therapy | 97 (3.1) | 86 (2.8) | 0.02 |
| Polymyxin B hemoperfusion | 868 (28.1) | 865 (27.8) | 0.01 |
| Central venous insertion | 1122 (36.4) | 1137 (36.5) | 0.003 |
| **Catecholamine**, *n* (%) |  |  |  |
| Dopamine | 2183 (70.8) | 2174 (69.8) | 0.02 |
| Noradrenaline | 1969 (63.8) | 1988 (63.9) | 0.001 |
| Vasopressin | 210 (6.8) | 193 (6.2) | 0.03 |
| Adrenaline | 167 (5.4) | 185 (5.9) | 0.02 |
| **Transfusion, *n* (%)** |  |  |  |
| Red cell transfusion | 1110 (36.0) | 1123 (36.1) | 0.002 |
| Platelets transfusion | 194 (6.3) | 198 (6.4) | 0.003 |
| Fresh frozen plasma transfusion | 823 (26.7) | 815 (26.2) | 0.01 |
| Antithrombin, *n* (%) | 721 (23.4) | 719 (23.1) | 0.01 |
| Recombinant human soluble thrombomodulin, *n* (%) | 586 (19.0) | 589.0 (18.9) | 0.002 |
| Immunoglobulin, *n* (%) | 771 (35.3) | 786 (35.8) | 0.01 |
| Albumin, *n* (%) | 1424 (65.2) | 1426 (65.0) | 0.004 |
| Danaparoid, *n* (%) | 26 (0.8) | 27.9 (0.9) | 0.01 |
| Low-molecular-weight heparin, *n* (%) | 35 (1.1) | 37.4 (1.2) | 0.01 |
| Hydrocortisone, *n* (%) | 310 (14.2) | 312 (14.2) | <0.001 |
| **Initial antibiotics use**, *n* (%) |  |  |  |
| Initial use of two or more | 935 (30.3) | 924 (29.7) | 0.01 |
| Penicillin | 0 (0) | 2 (0.1) | 0.04 |
| Ampicillin | 0 (0) | 2 (0.1) | 0.04 |
| Ampicillin/sulbactam | 79 (2.6) | 75 (2.4) | 0.01 |
| Piperacillin/tazobactam | 312 (10.1) | 309 (9.9) | 0.01 |
| First-generation cephalosporin | 133 (4.3) | 118 (3.8) | 0.03 |
| Second-generation cephalosporin | 1079.0 (35.0) | 1084 (34.8) | 0.003 |
| Third-generation cephalosporin without effect for *Pseudomonas aeruginosa* | 101 (3.3) | 98 (3.2) | 0.01 |
| Third-generation cephalosporin with effect for *Pseudomonas aeruginosa* | 12 (0.4) | 11 (0.4) | 0.01 |
| Fourth-generation cephalosporin | 76 (2.5) | 75 (2.4) | 0.003 |
| Carbapenem | 2025 (65.6) | 2059 (66.1) | 0.01 |
| Fluoroquinolone | 16 (0.5) | 18 (0.6) | 0.01 |
| Aminoglycoside | 37 (1.2) | 39 (1.2) | 0.01 |
| Clindamycin | 89 (4.1) | 89 (4.1) | 0.001 |
| Tetracycline | 6 (0.2) | 6 (0.2) | 0.002 |
| Macrolide | 0 (0) | 7 (0.2) | 0.07 |
| Anti-MRSA drug | 96 (3.1) | 84 (2.7) | 0.03 |
| Antifungal drug | 19 (0.6) | 18 (0.6) | 0.002 |

*Abbreviations*: H2RA, histamine-2 receptor antagonists; PPI, proton pump inhibiter SD, standard deviation; ICU, intensive care unit; IPTW, inverse probability of treatment weighting; HCU, high care unit; MRSA; methicillin-resistant *Staphylococcus aureus*; SMD, standardized mean difference.
