# Supplementary material for: Histamine-2 receptor antagonists versus proton pump inhibitors for septic shock after lower gastrointestinal tract perforation: a retrospective cohort study using a national inpatient database
Source: J Intensive Care. 2020 Jul 31;8:56. doi: 10.1186/s40560-020-00473-0 (PMC7395359; doi:10.1186/s40560-020-00473-0)
Supplement: Supplementary file 3 — Additional file 3: Additional Table 3. Risk differences for outcomes in the unmatched, propensity score-matched, and IPTW analysis groups. [file 40560_2020_473_MOESM3_ESM.docx]

**Additional Table 3: Risk differences for outcomes in the unmatched, propensity score-matched, and IPTW analysis groups.**

|  | **Unmatched groups** | **P** | **Propensity-Matched groups** | **P** | **IPTW analysis groups** | **P** |
| --- | --- | --- | --- | --- | --- | --- |
| Gastrointestinal bleeding requiring endoscopic hemostasis | −0.68% (−1.36% to 0.01%) | 0.076 | −0.55% (−1.39% to 0.29%) | 0.284 | −0.40% (−1.27% to 0.46%) | 0.361 |
| 28-day mortality | −1.86% (−4.14% to 0.42%) | 0.954 | −1.65% (−4.40% to 1.09%) | 0.386 | −0.46% (−3.04% to 2.12%) | 0.727 |
| *Clostridioides difficile* infection | 0.18% (−0.44% to 0.80%) | 0.663 | 0.18% (−0.44% to 0.81%) | 0.774 | 0.16% (−0.43% to 0.75%) | 0.598 |
| Hospital acquired pneumonia | −0.31% (−1.58% to 0.96%) | 0.681 | −1.29% (−2.87% to 0.29%) | 0.138 | −0.60% (−1.95% to 0.75%) | 0.385 |

*Abbreviation*: IPTW, inverse probability of treatment weighting.
